# Supplementary material for: Developing a video expert panel as a reference standard to evaluate respiratory rate counting in paediatric pneumonia diagnosis: protocol for a cross-sectional study
Source: BMJ Open. 2022 Nov 15;12(11):e067389. doi: 10.1136/bmjopen-2022-067389 (PMC9668034; doi:10.1136/bmjopen-2022-067389)
Supplement: Author's manuscript [file bmjopen-2022-067389.draft_revisions.pdf]

BMJ Open is committed to open peer review. As part of this commitment we make the peer review history of every article we publish publicly available.

When an article is published we post the peer reviewers' comments and the authors' responses online. We also post the versions of the paper that were used during peer review. These are the versions that the peer review comments apply to.

The versions of the paper that follow are the versions that were submitted during the peer review process. They are not the versions of record or the final published versions. They should not be cited or distributed as the published version of this manuscript.

BMJ Open is an open access journal and the full, final, typeset and author-corrected version of record of the manuscript is available on our site with no access controls, subscription charges or pay-per-view fees (http://bmjopen.bmj.com).

If you have any questions on BMJ Open's open peer review process please email info.bmjopen@bmj.com

BMJ Open

Developing a video expert panel as a reference standard to evaluate respiratory rate counting in paediatric pneumonia diagnosis: protocol for a cross-sectional study

Journal:	*BMJ Open*
Manuscript ID	bmjopen-2022-067389
Article Type:	Protocol
Date Submitted by the Author:	10-Aug-2022

Complete List of Authors:	Khan, Ahad; The University of Edinburgh, Usher Institute; Projahnmo Research Foundation,<br>Ahmed, Salahuddin; Projahnmo Research Foundation; The University of Edinburgh, Usher Institute<br>Chowdhury, Nabidul Haque; Projahnmo Research Foundation<br>Islam, Md Shafiqul; Projahnmo Research Foundation<br>McCollum, Eric D.; Johns Hopkins School of Medicine; Johns Hopkins University Bloomberg School of Public Health, Department of International Health<br>King, Carina; Karolinska Institute, Department of Global Public Health<br>Shi, Ting; The University of Edinburgh, Usher Institute<br>Nahar, Kamrun; Shaheed Suhrawardi Medical College Hospital, Department of Paediatrics<br>Simpson, Robynne; Royal Hospital for Children<br>Ahmed, Ayaz; Royal Hospital for Children<br>Rahman, Md Mozibur; Institute of Child and Mother Health, Department of Neonatology<br>Baqui, Abdullah; Johns Hopkins University Bloomberg School of Public Health, Department of International Health<br>Cunningham, Steven; The University of Edinburgh Centre for Inflammation Research, Department of Paediatric Respiratory Medicine<br>Campbell, Harry; The University of Edinburgh, Usher Institute

Keywords:	Community child health < PAEDIATRICS, PRIMARY CARE, Public health < INFECTIOUS DISEASES

SCHOLARONE™
Manuscripts

Developing a video expert panel as a reference standard to evaluate respiratory rate counting in paediatric pneumonia diagnosis: protocol for a cross-sectional study

Ahad Mahmud Khan[1,2], Salahuddin Ahmed[1,2], Nabidul Haque Chowdhury[2], Md Shafiqul Islam[2], Eric D McCollum[3,4], Carina King[5], Ting Shi[1], Kamrun Nahar[6], Robynne Simpson[7], Ayaz Ahmed[7], Md Mozibur Rahman[8], Abdullah H Baqui[4], Steven Cunningham[9], Harry Campbell[1], RESPIRE Collaboration

[1]Usher Institute, College of Medicine and Veterinary Medicine, University of Edinburgh, UK

[2]Projahnmo Research Foundation, Dhaka, Bangladesh

[3]Eudowood Division of Paediatric Respiratory Sciences, Department of Paediatrics, School of Medicine, Johns Hopkins University, Baltimore, Maryland, USA

[4]Department of International Health, Johns Hopkins Bloomberg School of Public Health, Baltimore, Maryland, USA

[5]Department of Global Public Health, Karolinska Institutet, Stockholm, Sweden

[6]Department of Paediatrics, Shaheed Suhrawardi Medical College Hospital, Dhaka, Bangladesh

[7]Royal Hospital for Children, Glasgow, UK

[8]Department of Neonatology, Institute of Child and Mother Health, Dhaka, Bangladesh

[9]Centre for Inflammation Research, University of Edinburgh, UK

Corresponding Author:

Ahad Mahmud Khan

The University of Edinburgh, Edinburgh, UK

Email: ahad_mahmud@hotmail.com, A.M.Khan@sms.ed.ac.uk

Preprint: https://doi.org/10.1101/2022.08.08.22278556

For peer review only

For peer review only - http://bmjopen.bmj.com/site/about/guidelines.xhtml

Abstract

Introduction

Manual counting of respiratory rate (RR) in children is challenging for health workers and can result in misdiagnosis of pneumonia. Some novel RR counting devices automate the counting of RR and classification of fast breathing. The absence of an appropriate reference standard to evaluate the performance of these devices is a challenge. If good quality videos could be captured, with RR interpretation from these videos systematically conducted by an expert panel, it could act as a reference standard. This study is designed to develop a video expert panel (VEP) as a reference standard to evaluate RR counting for identifying pneumonia in children.

Methods and analysis

Using a cross-sectional design, we will enrol children aged 0-59 months presenting with suspected pneumonia at different levels of health facilities in Dhaka and Sylhet, Bangladesh. We will video record a physician/health worker counting RR manually and also using an automated RR counter (ChARM) from each child. We will establish a standard operating procedure for capturing quality videos, make a set of reference videos, and train and standardise the VEP members using the reference videos. After that, we will assess the performance of the VEP as a reference standard to evaluate RR counting. We will calculate the mean difference and proportions of agreement within ±2 breaths per minute and create Bland-Altman plots with limits of agreement between VEP members.

Ethics and dissemination

The study protocol was approved by the National Research Ethics Committee of Bangladesh Medical Research Council (BMRC), Bangladesh (registration number: 39315022021) and Edinburgh Medical School Research Ethics Committee (EMREC), Edinburgh, UK (REC Reference: 21-EMREC-040). Dissemination of the study findings will be through conference presentations and publications in peer-reviewed scientific journals.

Keywords

Pneumonia, respiratory rate, fast breathing, reference standard, video expert panel

Word count: 2771

For peer review only - http://bmjopen.bmj.com/site/about/guidelines.xhtml

Strengths and limitations of this study

- Expert paediatricians will provide feedback to develop a standard operating procedure for videography of child chest movements.
- The video expert panel will be trained and standardized using the expert paediatrician-interpreted reference videos.
- Video expert panel members will be masked to respiratory rate counted by each other and to respiratory rate manual counts, and with the automated counter.
- Children with varying severity of illness will be enrolled from different levels of health facilities in Bangladesh.
- Despite the availability of multiple respiratory rate counters, only the ChARM device will be used in this study.

For peer review only

For peer review only - http://bmjopen.bmj.com/site/about/guidelines.xhtml

Introduction

Pneumonia is one of the leading causes of mortality in children aged below five years worldwide.[1] Approximately 68 million pneumonia episodes and 0.65 million deaths occur annually in under-five children due to pneumonia.[2] The highest number of deaths due to pneumonia among children below five years were in sub-Saharan Africa, South Asia, and Southeast Asia. There were an estimated 2.7 million episodes of pneumonia in Bangladesh and 21,275 deaths due to childhood pneumonia in 2015.[3]

According to the World Health Organization (WHO) guidelines, pneumonia diagnosis in children for frontline health workers is primarily based on increased respiratory rate (RR) and/or chest indrawing.[4 5] Fast breathing is the most common sign of pneumonia. It is most commonly identified by counting the RR manually.[5] However, manual counting can be challenging for the health workers[6], often leading to incorrect diagnosis and, consequently, inappropriate treatment.[7-9]

Better performing RR diagnostics to support frontline health workers might improve the diagnosis of pneumonia. Three automated RR counting devices are considered suitable for use by health workers, i.e., Children's Automated Respiration Monitor (ChARM)[10 11], Rad-G[12 13] and uPM60[14]. The Philips ChARM converts chest movements detected by accelerometers into a precise breathing count using specially designed algorithms. The device is placed around the child's belly, and it automatically counts RR and classifies fast breathing according to WHO guidelines[10 11]. The usability and acceptability of this device have been tested in a study in Ethiopia[15] and Nepal.[16]

A reference standard is essential to evaluate the performance of RR counters in field settings. However, the absence of an appropriate reference standard is a challenge. Different reference standards have been used in various studies.[17 18] Most of the existing studies have used manual RR count by an expert person (e.g., physician, nurse) as the reference standard.[19] Few studies have been found using automated monitors.[20 21] The possible biases using a human expert's count as the reference standard include difficulty measuring the RR over the same simultaneous period and inconsistencies in human expert RR counting.[6] The automated monitors do not measure chest movements directly, but other variables such as carbon dioxide (CO_2), pulse oximeter signal or photoplethysmogram (PPG), sound etc., are used to extract RR.[19 22-24]

Videography of a child's chest movements and interpretation by the experts could be used as a reference standard.[17 18 25] If quality videos could be recorded and the interpretation of RR from these videos could be systematically conducted by a video expert panel (VEP), it could be an ideal and non-biased reference standard. This study aims to develop a VEP as a reference standard for evaluating RR counting manually and using ChARM in paediatric pneumonia diagnosis.

Study objectives

Primary objectives:

1. To develop a standard operating procedure (SOP) for video recording a child's chest movements for RR interpretation.
2. To create a set of reference videos for the training and standardisation of the VEP members.

For peer review only - http://bmjopen.bmj.com/site/about/guidelines.xhtml

3. To train and standardise the VEP members to interpret RR using the reference videos.
4. To assess the performance of the VEP to evaluate RR counting manually and with ChARM based on videos captured in real-world settings.

Secondary objectives:

1. To assess the accuracy of the ChARM device in counting RR compared to the VEP as the reference.
2. To determine the duration of counting RR using the ChARM device.
3. To explore the potential influence of the ChARM device on RR count compared to standard observation techniques.

Methods and analysis

Study design and settings

This will be a cross-sectional study. First, we will capture videos of child chest movements, develop a SOP of videography, make a set of reference videos and use these reference videos to train and standardize the VEP. We will record these videos from the inpatient department (IPD) of the Institute of Child and Mother Health (ICMH), Dhaka, Bangladesh. After that, we will use the VEP as a reference standard to evaluate RR counting. The videos will be recorded from different levels of health facilities in Bangladesh. Three community clinics (CCs) and a sub-district hospital (Zakiganj Upazila Health Complex) in Sylhet and ICMH in Dhaka will be selected. CCs are the lower-level health facility in Bangladesh staffed by community healthcare providers (CHCP).[26] Patients from the CCs are referred to sub-district hospitals. Patients often go to sub-district hospitals directly.[27]

Study population

Inclusion criteria:

i. Age <2 months presenting with any illness
ii. Age 2-59 months presenting with cough and/or difficulty breathing

Exclusion criteria:

i. Presence of any danger sign (unable to drink or feed, vomit everything, convulsions, lethargic or unconscious)
ii. Unwilling to provide written informed consent

Study procedures

Enrolment and consenting:

In the hospital, a physician will screen all children who will visit the outpatient clinic (sub-district hospital) or are already admitted to the inpatient department (ICMH) and enrol eligible children. In the CC, the CHCP will screen every child who will visit his/her CC. If a child becomes eligible, the CHCP will enrol the child and contact research staff who will visit the CC immediately and will complete data collection. Informed written consent will be obtained from the child's mother, father, or other available caregiver before data collection.

For peer review only - http://bmjopen.bmj.com/site/about/guidelines.xhtml

Developing SOP of videography:

Research staff will record videos of child chest movements from 30 children. The Canon EOS M50 camera[28] will be used for recording videos. If a good source of natural light is not available, we will use a light-emitting diode (LED) lamp for lighting. The child's clothes will be removed from the lower end of the neck through the umbilicus so that the chest and belly are exposed. The child's face or any other identifiable part will not be recorded. The videos will be recorded from different camera positions and angles with varying levels of light. The shots will be taken either using a tripod or not using it. Video recording will be done both when counting RR manually twice and using the ChARM device twice.

Once the child is calm, the research staff will start video recording, and the physician will tap the microphone with his/her finger and begin counting RR. The physician will count RR for one minute and tap the microphone again when counting is finished, and the research staff will stop the recording. After that, the physician will attach the ChARM device, and the research staff will start video recording. Once the child is calm, the physician will start the ChARM device and also begin counting RR manually, and the video recording will be done in parallel to the physician's use of ChARM like before. The physician will count the total number of breaths until ChARM completes its counting. The ChARM device will show a green or red signal when it finishes its count. The video recording will continue until the device is done. The time taken by the ChARM device to get the RR will be recorded. A maximum of 3 minutes will be allowed for the ChARM to register a reading or display an error message. Otherwise, it will be documented as a failed attempt.

Each video file will be saved in the camera with a file name. The research staff will note the file name in the data collection sheet. Then the videos will be downloaded from the camera and saved on a password-protected computer. After that he will edit the video by removing additional portions of the recording, removing sound or identifiable features, and reducing file size. Video Editing Software (Adobe Premiere Pro) will be used for video editing.

There will be two expert paediatricians (i.e., medical practitioners having a post-graduate degree in paediatrics and specialized in treating sick children). These videos will be sent to them for their feedback on video quality (decision of using tripod, camera positions, angles, light, or other issues) and to count RR. Their feedback will be incorporated, and the video recording procedures will be modified accordingly. A SOP for videography will be developed to maintain consistency across all study hospitals/clinics.

Figure 1 shows the schematic diagram of developing the SOP for videography.

Making a set of reference videos:

Following the development of the SOP, videos will be recorded from 50 children using this standardised procedure. A physician will count the RR manually once and with the ChARM device once, and video recording will be done simultaneously. The same procedure described in Figure 1 will be followed. If the physician fails in their first attempt to have a satisfactory recording, they will make two more attempts for each manual and ChARM counts. The reason for the attempted failure will be documented. The condition of the child during RR measurement will also be noted.

For peer review only - http://bmjopen.bmj.com/site/about/guidelines.xhtml

There will be three expert paediatricians. Each video will randomly be allocated to two of the paediatricians. If both paediatricians disagree, i.e., disagreement in readability or difference of RR >±2 breaths per minute (bpm), then the video will be sent to the third paediatrician. If any two paediatricians agree, i.e., agreement in interpretability and difference of RR ≤±2 bpm, the videos will be considered reference videos. The mean RR count of paediatricians in agreement will be considered as the final RR count.

Figure 2 presents the schematic diagram of making a set of reference videos.

Training and standardisation of VEP members:

Six local physicians with a MBBS degree will be invited for training and standardisation. The physicians will be trained on counting RR using videos of known RR counts from reference videos. The Principal Investigator (PI) will conduct the training sessions. After completion of training, they will be evaluated individually in a blinded manner. Twenty videos will be assigned to each VEP member from the reference videos. Those physicians who achieve a pass mark (at least 80%) in the evaluation will be considered to work as VEP members in the study.

Evaluating the performance of VEP:

The performance of VEP will be evaluated using the videos recorded in different levels of health facilities (CCs, sub-district hospital and ICMH). RR of each child will be assessed twice, i.e., manually and using ChARM by the CHCP in the CC and by a physician in the hospital (as described in Figure 1). The videos will be recorded following the SOP. Each video will be randomly assigned to two VEP members to interpret the RR. If there is an agreement of being uninterpretable, the videos will be excluded. If the RR is ≤±2 bpm between both members, their average RR will be considered final. However, if there is a disagreement in interpretability or the difference of the RR count is >2 bpm between the first two members, the video will be sent to a third-panel member. If there are inconsistencies among all three members (if the RR is not ≤2 bpm between any two members), the videos will be sent to a fourth-panel member. If there is agreement between the fourth-panel member and any other member who read the video earlier, their average RR will be considered final. However, if there is no agreement between any two panel members, the videos will be excluded from the analysis (Figure 3).

Quality control measures:

The videos will be checked periodically to ensure quality by the PI. Any quality issues will be reported, and corrective feedback will be provided. The expert paediatricians will view a 10% random sample of videos in which they are blinded to the panel's final classification. The panel's overall performance will be evaluated using the expert's interpretation as the reference. Lastly, to monitor individual member interpretation reproducibility, all members will randomly be reassigned 20% of images they interpreted previously to check intra-reader agreement.

Development of a web-based automated system:

A web-based automated system will be developed. This system will use the Hypertext Preprocessor (PHP) platform for the user interface and the Structured Query Language (SQL) platform for the database. The purpose of developing this system is an automated distribution

For peer review only - http://bmjopen.bmj.com/site/about/guidelines.xhtml

of the video recordings among the expert paediatricians and VEP members, the input of readings by the members and the generation of automated reports. The system will be password-protected and connected to a secured server. The expert paediatricians and VEP members will use this program. Each will need to log in with their user ID and password. Access to each expert paediatrician and VEP member will be limited to what they are assigned. When logged in, they will see the list of recordings assigned to them. They will open the video on the computers and interpret the RR. The VEP members will be blinded to other members' readings.

Operational definitions

Interpretability:

The video will be considered 'interpretable' when the expert paediatrician or VEP member will be able to view the child's chest movements and measure RR for the whole duration. On the other hand, a video will be counted as 'uninterpretable' if the physician will not be able to view and count RR for the whole duration of the recording. The videos can be unreadable for various reasons, e.g., wrong position and angle of the camera, inappropriate focus, poor lighting, inadequate exposure or if the child is agitated, moving, crying etc.

Agreement and disagreement:

The agreement will be defined when two expert paediatricians or VEP members interpret the video as

- uninterpretable or
- interpretable and the difference between RR count ≤±2 bpm.

Disagreement is when

- one expert paediatrician/VEP member interprets the video as interpretable, and another interprets it as unreadable or
- the difference between their RR count is >2 bpm.

Data collection and storage

Field data will be collected in paper forms. After entry, data will be transferred to a password-protected server (SQL Server 2008 R2) located in Dhaka, Bangladesh, in real-time using internet connectivity. Recorded videos will be transferred to encrypted OneDrive/Dropbox cloud storage. The VEP members will interpret RR from recorded videos using the web-based automated software, and the data will also be stored on the server in real-time. De-identified data will be transferred and stored on the DataShare server at the University of Edinburgh, UK. Data collection started on 6 December 2021 and is planned to be completed by October 2022.

Sample size calculation

The videos from the first 30 children will be used to establish the best procedure for capturing videos. After that, the videos from 50 children will be used for making a set of reference videos to train and standardize VEP. To evaluate the performance of VEP, Bland-Altman's statistical methods for assessing agreement between two methods of clinical measurement are used.[29] [30] A total of 226 interpretable videos of each method (manual and ChARM) are required, which will provide 90% power to assess agreement in RR counts between two VEP members, considering type-I error α=0.05, expected mean difference ±0.5, expected SD 1.5 and

For peer review only - http://bmjopen.bmj.com/site/about/guidelines.xhtml

maximum allowed difference 4. Assuming 80% interpretability of the videos and 20% failure to record videos, the required number of children is about 350.

Statistical analysis

To standardize the VEP members, we will estimate the mean difference of RR counts and the percent agreement within ±2bpm between each VEP member and expert paediatricians. We will consider expert paediatricians' counts as the gold standard. To assess the performance of VEP members, we will produce Bland-Altman plots[29] to assess the agreement in RR counts and calculate the percent agreement between primary panel members. We will also assess the intra-reader agreement of each VEP member. We will use Cohen's kappa statistic to estimate the inter-reader agreement of identifying fast breathing between panel members. We will also measure the mean difference of RR counts and the percent agreement within ±2bpm between each VEP member and expert paediatricians from QC data.

Ethics and dissemination

Ethical approval was obtained from the National Research Ethics Committee of Bangladesh Medical Research Council (BMRC), Bangladesh (Registration Number: 39315022021), and Edinburgh Medical School Research Ethics Committee (EMREC), Edinburgh, UK (REC Reference: 21-EMREC-040). Informed written consent will be taken from the parent or guardian of each child. Dissemination will be through conference presentations and publications in peer-reviewed journals. Anonymised data files will also be stored securely in the DataStore repository at the University of Edinburgh, UK and will be shared after the publication the main paper.

Acknowledgments

The authors are grateful for the support and contributions of Dr Arunangshu Dutta Roy, Asim Nehal, Rizouan Ur Rashid, Rakib Bhuiyan, Dr Nusrat Sharmin Asma, Dr Farjana Hossain, Dr Muhammad Shariful Islam, Dr Md. Jahurul Islam, and the Ministry of Health and Family Welfare, Government of Bangladesh.

Footnotes

Author Contributions

AMK and HC conceptualised and designed this study. HC, SC, AHB, EDM and TS provided mentorship to AMK. HC, SC and EDM critically reviewed the study design. KN, RMS and AA will review the videos and provide feedback to video quality. NHC and MSI will be responsible for data management. AMK drafted the manuscript, and all authors critically reviewed and approved the final manuscript before submission. The RESPIRE collaboration comprises the UK Grant holders, Partners and research teams as listed on the RESPIRE website (www.ed.ac.uk/usher/respire) including Linda Bauld.

Funding

This research was funded by the UK National Institute for Health Research (NIHR) (Global Health Research Unit on Respiratory Health (RESPIRE); 16/136/109) using UK aid from the UK Government to support global health research. The views expressed in this publication are those of the author(s) and not necessarily those of the NIHR or the UK Government.

Competing interests

None declared.

For peer review only - http://bmjopen.bmj.com/site/about/guidelines.xhtml

Patient and public involvement

Patients and/or the public were not involved in this research's design, conduct, reporting, or dissemination plans.

References

1. Liu L, Oza S, Hogan D, et al. Global, regional, and national causes of under-5 mortality in 2000-15: an updated systematic analysis with implications for the Sustainable Development Goals. *Lancet* 2016;388(10063):3027-35. doi: 10.1016/s0140-6736(16)31593-8 [published Online First: 2016/11/15]
2. GBD 2016. Estimates of the global, regional, and national morbidity, mortality, and aetiologies of lower respiratory infections in 195 countries, 1990-2016: a systematic analysis for the Global Burden of Disease Study 2016. *Lancet Infect Dis* 2018;18(11):1191-210. doi: 10.1016/s1473-3099(18)30310-4 [published Online First: 2018/09/24]
3. GBD 2015. Estimates of the global, regional, and national morbidity, mortality, and aetiologies of lower respiratory tract infections in 195 countries: a systematic analysis for the Global Burden of Disease Study 2015. *Lancet Infect Dis* 2017;17(11):1133-61. doi: 10.1016/s1473-3099(17)30396-1 [published Online First: 2017/08/28]
4. World Health Organization. IMCI information package. Integrated Management of Childhood Illness (IMCI). Geneva, 1999.
5. World Health Organization. Integrated Management of Childhood Illness: Chart Booklet. *World Health Organization: Geneva* 2014
6. Khan AM, O'Donald A, Shi T, et al. Accuracy of non-physician health workers in respiratory rate measurement to identify paediatric pneumonia in low-and middle-income countries: A systematic review and meta-analysis. *J Glob Health* 2022;12
7. Kallander K, Tomson G, Nsabagasani X, et al. Can community health workers and caretakers recognise pneumonia in children? Experiences from western Uganda. *Transactions of the Royal Society of Tropical Medicine and Hygiene* 2006;100(10):956-63. doi: 10.1016/j.trstmh.2005.11.004 [published Online First: 2006/02/04]
8. Mukanga D, Babirye R, Peterson S, et al. Can lay community health workers be trained to use diagnostics to distinguish and treat malaria and pneumonia in children? Lessons from rural Uganda. *Tropical medicine & international health : TM & IH* 2011;16(10):1234-42. doi: 10.1111/j.1365-3156.2011.02831.x [published Online First: 2011/07/15]
9. Miller NP, Amouzou A, Tafesse M, et al. Integrated community case management of childhood illness in Ethiopia: implementation strength and quality of care. *The American journal of tropical medicine and hygiene* 2014;91(2):424-34. doi: 10.4269/ajtmh.13-0751 [published Online First: 2014/05/07]
10. Shah R, Jenda G, Lwesha V, et al. An integrated diagnostic device for neonatal sepsis and childhood pneumonia. *Journal of public health in Africa* 2018;9(1):780. doi: 10.4081/jphia.2018.780 [published Online First: 2018/08/07]
11. Philips. Philips Introduces New Diagnostic Device to Help Prevent Childhood Pneumonia in Low-resource Countries 2019 [Available from: file:///C:/Users/Ahad/Downloads/Backgrounder%20-%20Philips%20Children%E2%80%99s%20Automated%20Respiration%20Monitor%20(1).pdf accessed 18 November 2019.
12. Masimo. Masimo Introduces Rad-G™ Pulse Oximeter 2017 [Available from: http://masimocorp.gcs-web.com/news-releases/news-release-details/masimo-introduces-rad-gtm-pulse-oximeter accessed viewed 07 November 2018.
13. Trademarkia. RAD-G Trademark Information 2017 [Available from: https://www.trademarkia.com/radg-87345900.html accessed 07 November 2018.

For peer review only - http://bmjopen.bmj.com/site/about/guidelines.xhtml

14. Utech. uPM60 Handheld Vital Signs Monitor 2020 [Available from: http://www.chinautech.com/cn/col.jsp?id=161 accessed 10 May 2020.

15. Ward C, Baker K, Smith H, et al. Usability and acceptability of an automated respiratory rate counter to assess children for symptoms of pneumonia: a prospective, cohort study in Ethiopia. *Acta paediatrica (Oslo, Norway : 1992)* 2019 doi: 10.1111/apa.15074 [published Online First: 2019/10/23]

16. Källander K, Ward C, Smith H, et al. Usability and acceptability of an automated respiratory rate counter to assess childhood pneumonia in Nepal. *Acta paediatrica (Oslo, Norway : 1992)* 2020;109(6):1207-20. doi: 10.1111/apa.15108 [published Online First: 2019/11/26]

17. Selecting a reference standard for evaluating respiratory rate devices to diagnose symptoms of pneumonia in children under 5: Lessons learned from resource-poor settings in Sub-Saharan Africa and Asia. American Journal of Tropical Medicine and Hygiene; 2017. Amer Soc Trop Med & Hygiene 8000 Westpart Dr, Ste 130, Mclean, VA 22101 USA.

18. Ansermino JM, Dumont G, Ginsburg AS. How Uncertain Is Our Reference Standard for Respiratory Rate Measurement? *Am J Respir Crit Care Med* 2019;199(8):1036-37. doi: 10.1164/rccm.201812-2266LE [published Online First: 2019/01/24]

19. Ginsburg AS, Lenahan JL, Izadnegahdar R, et al. A Systematic Review of Tools to Measure Respiratory Rate in Order to Identify Childhood Pneumonia. *Am J Respir Crit Care Med* 2018;197(9):1116-27. doi: 10.1164/rccm.201711-2233CI [published Online First: 2018/02/24]

20. Baker K, Alfven T, Mucunguzi A, et al. Performance of Four Respiratory Rate Counters to Support Community Health Workers to Detect the Symptoms of Pneumonia in Children in Low Resource Settings: A Prospective, Multicentre, Hospital-Based, Single-Blinded, Comparative Trial. *EClinicalMedicine* 2019;12:20-30. doi: 10.1016/j.eclinm.2019.05.013 [published Online First: 2019/08/08]

21. Becker C, Achermann S, Rocque M, et al. Camera-based measurement of respiratory rates is reliable. *Eur J Emerg Med* 2018;25(6):416-22. doi: 10.1097/mej.0000000000000476 [published Online First: 2017/06/03]

22. Charlton PH, Bonnici T, Tarassenko L, et al. An assessment of algorithms to estimate respiratory rate from the electrocardiogram and photoplethysmogram. *Physiol Meas* 2016;37(4):610-26. doi: 10.1088/0967-3334/37/4/610 [published Online First: 2016/03/31]

23. Li W, Liu J, Yu L. [Study on Respiratory Rate Monitoring Method Based on Breath Sounds]. *Zhongguo Yi Liao Qi Xie Za Zhi* 2018;42(6):391-94. doi: 10.3969/j.issn.1671-7104.2018.06.001 [published Online First: 2018/12/19]

24. Daw W, Kingshott R, Saatchi R, et al. Medical devices for measuring respiratory rate in children: a review. *Journal of Advances in Biomedical Engineering and Technology* 2016;3:21-27.

25. Stratil AS, Ward C, Habte T, et al. Evaluating the Interrater Agreement and Acceptability of a New Reference Tool for Assessing Respiratory Rate in Children under Five with Cough and/or Difficulty Breathing. *J Trop Pediatr* 2021;67(2) doi: 10.1093/tropej/fmab046

26. Karim RM, Abdullah MS, Rahman AM, et al. Identifying role of perceived quality and satisfaction on the utilization status of the community clinic services; Bangladesh context. *BMC Health Serv Res* 2016;16:204. doi: 10.1186/s12913-016-1461-z [published Online First: 2016/06/28]

27. Ahmed S, Siddique AK, Iqbal A, et al. Causes for hospitalizations at upazila health complexes in Bangladesh. *J Health Popul Nutr* 2010;28(4):399-404. doi: 10.3329/jhpn.v28i4.6047 [published Online First: 2010/09/10]

28. Canon. Canon EOS M50 [Available from: https://www.canon-europe.com/cameras/eos-m50/ accessed 21 June 2021 2020.

For peer review only - http://bmjopen.bmj.com/site/about/guidelines.xhtml

29. Bland JM, Altman DG. Statistical methods for assessing agreement between two methods of clinical measurement. *Lancet* 1986;1(8476):307-10.
30. Lu MJ, Zhong WH, Liu YX, et al. Sample Size for Assessing Agreement between Two Methods of Measurement by Bland-Altman Method. *Int J Biostat* 2016;12(2) doi: 10.1515/ijb-2015-0039 [published Online First: 2016/11/14]

Figure 1: Developing SOP of videography

Figure 2: Making a set of reference videos

Figure 3: Respiratory rate interpretation from video recording by video expert panel

For peer review only

For peer review only - http://bmjopen.bmj.com/site/about/guidelines.xhtml

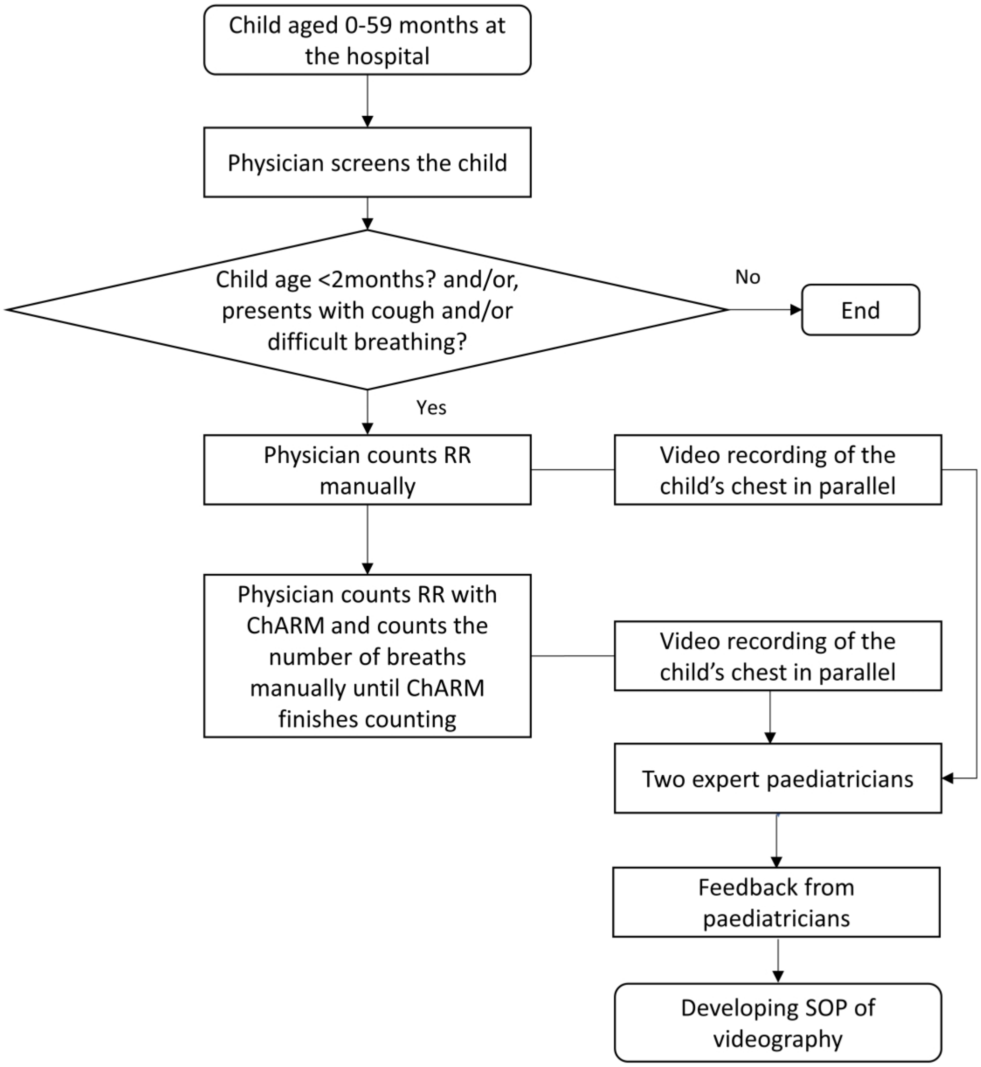

Developing SOP of videography

456x497mm (236 x 236 DPI)

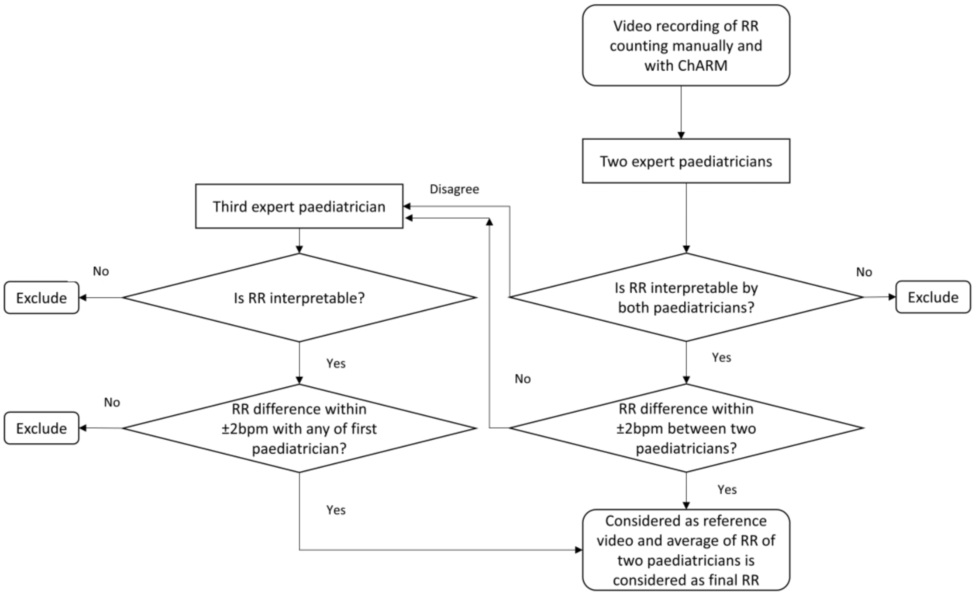

Making a set of reference videos

719x433mm (236 x 236 DPI)

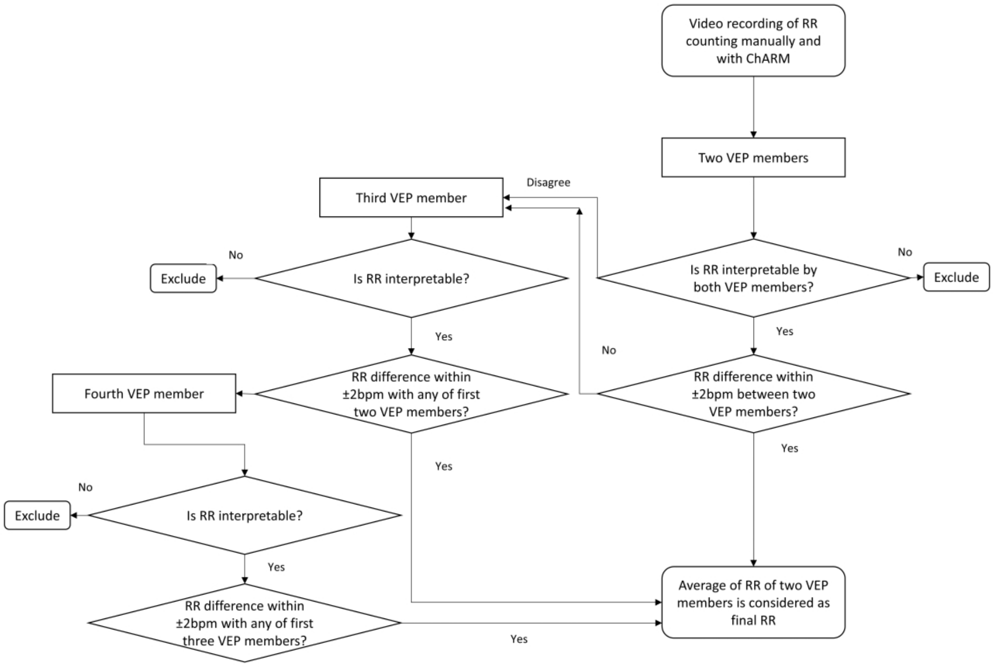

Respiratory rate interpretation from video recording by video expert panel

813x550mm (236 x 236 DPI)

STROBE Statement—Checklist of items that should be included in reports of ***cross-sectional studies***

	Item No	Recommendation	Page No
**Title and abstract**	1	(*a*) Indicate the study's design with a commonly used term in the title or the abstract	1, 2
		(*b*) Provide in the abstract an informative and balanced summary of what was done and what was found	2
**Introduction**			
Background/rationale	2	Explain the scientific background and rationale for the investigation being reported	4
Objectives	3	State specific objectives, including any prespecified hypotheses	4,5
**Methods**			
Study design	4	Present key elements of study design early in the paper	5
Setting	5	Describe the setting, locations, and relevant dates, including periods of recruitment, exposure, follow-up, and data collection	5, 8
Participants	6	(*a*) Give the eligibility criteria, and the sources and methods of selection of participants	5
Variables	7	Clearly define all outcomes, exposures, predictors, potential confounders, and effect modifiers. Give diagnostic criteria, if applicable	8
Data sources/ measurement	8*	For each variable of interest, give sources of data and details of methods of assessment (measurement). Describe comparability of assessment methods if there is more than one group	*5-8*
Bias	9	Describe any efforts to address potential sources of bias	N/A
Study size	10	Explain how the study size was arrived at	8-9
Quantitative variables	11	Explain how quantitative variables were handled in the analyses. If applicable, describe which groupings were chosen and why	9
Statistical methods	12	(*a*) Describe all statistical methods, including those used to control for confounding	9
		(*b*) Describe any methods used to examine subgroups and interactions	9
		(*c*) Explain how missing data were addressed	N/A
		(*d*) If applicable, describe analytical methods taking account of sampling strategy	8
		(*e*) Describe any sensitivity analyses	N/A
**Results**			
Participants	13*	(a) Report numbers of individuals at each stage of study—eg numbers potentially eligible, examined for eligibility, confirmed eligible, included in the study, completing follow-up, and analysed	N/A
		(b) Give reasons for non-participation at each stage	N/A
		(c) Consider use of a flow diagram	N/A
Descriptive data	14*	(a) Give characteristics of study participants (eg demographic, clinical, social) and information on exposures and potential confounders	N/A
		(b) Indicate number of participants with missing data for each variable of interest	N/A
Outcome data	15*	Report numbers of outcome events or summary measures	N/A
Main results	16	(*a*) Give unadjusted estimates and, if applicable, confounder-adjusted estimates and their precision (eg, 95% confidence interval). Make clear which confounders were adjusted for and why they were included	N/A

For peer review only

For peer review only - http://bmjopen.bmj.com/site/about/guidelines.xhtml

		(*b*) Report category boundaries when continuous variables were categorized	N/A
		(*c*) If relevant, consider translating estimates of relative risk into absolute risk for a meaningful time period	N/A
Other analyses	17	Report other analyses done—eg analyses of subgroups and interactions, and sensitivity analyses	N/A
**Discussion**			
Key results	18	Summarise key results with reference to study objectives	N/A
Limitations	19	Discuss limitations of the study, taking into account sources of potential bias or imprecision. Discuss both direction and magnitude of any potential bias	N/A
Interpretation	20	Give a cautious overall interpretation of results considering objectives, limitations, multiplicity of analyses, results from similar studies, and other relevant evidence	N/A
Generalisability	21	Discuss the generalisability (external validity) of the study results	N/A
**Other information**			
Funding	22	Give the source of funding and the role of the funders for the present study and, if applicable, for the original study on which the present article is based	9

*Give information separately for exposed and unexposed groups.

Note: An Explanation and Elaboration article discusses each checklist item and gives methodological background and published examples of transparent reporting. The STROBE checklist is best used in conjunction with this article (freely available on the Web sites of PLoS Medicine at http://www.plosmedicine.org/, Annals of Internal Medicine at http://www.annals.org/, and Epidemiology at http://www.epidem.com/). Information on the STROBE Initiative is available at www.strobe-statement.org.

For peer review only

For peer review only - http://bmjopen.bmj.com/site/about/guidelines.xhtml

BMJ Open

Developing a video expert panel as a reference standard to evaluate respiratory rate counting in paediatric pneumonia diagnosis: protocol for a cross-sectional study

Journal:	*BMJ Open*
Manuscript ID	bmjopen-2022-067389.R1
Article Type:	Protocol
Date Submitted by the Author:	10-Oct-2022
Complete List of Authors:	Khan, Ahad; The University of Edinburgh, Usher Institute; Projahnmo Research Foundation,<br>Ahmed, Salahuddin; Projahnmo Research Foundation; The University of Edinburgh, Usher Institute<br>Chowdhury, Nabidul Haque; Projahnmo Research Foundation<br>Islam, Md Shafiqul; Projahnmo Research Foundation<br>McCollum, Eric D.; Johns Hopkins School of Medicine; Johns Hopkins University Bloomberg School of Public Health, Department of International Health<br>King, Carina; Karolinska Institute, Department of Global Public Health<br>Shi, Ting; The University of Edinburgh, Usher Institute<br>Nahar, Kamrun; Shaheed Suhrawardi Medical College Hospital, Department of Paediatrics<br>Simpson, Robynne; Royal Hospital for Children<br>Ahmed, Ayaz; Royal Hospital for Children<br>Rahman, Md Mozibur; Institute of Child and Mother Health, Department of Neonatology<br>Baqui, Abdullah; Johns Hopkins University Bloomberg School of Public Health, Department of International Health<br>Cunningham, Steven; The University of Edinburgh Centre for Inflammation Research, Department of Paediatric Respiratory Medicine<br>Campbell, Harry; The University of Edinburgh, Usher Institute
**Primary Subject Heading**:	Paediatrics
Secondary Subject Heading:	Public health, Global health
Keywords:	Community child health < PAEDIATRICS, PRIMARY CARE, Public health < INFECTIOUS DISEASES

SCHOLARONE™
Manuscripts

Developing a video expert panel as a reference standard to evaluate respiratory rate counting in paediatric pneumonia diagnosis: protocol for a cross-sectional study

Ahad Mahmud Khan[1,2], Salahuddin Ahmed[1,2], Nabidul Haque Chowdhury[2], Md Shafiqul Islam[2], Eric D McCollum[3,4], Carina King[5], Ting Shi[1], Kamrun Nahar[6], Robynne Simpson[7], Ayaz Ahmed[7], Md Mozibur Rahman[8], Abdullah H Baqui[4], Steven Cunningham[9], Harry Campbell[1], RESPIRE Collaboration

[1]Usher Institute, College of Medicine and Veterinary Medicine, University of Edinburgh, UK

[2]Projahnmo Research Foundation, Dhaka, Bangladesh

[3]Eudowood Division of Paediatric Respiratory Sciences, Department of Paediatrics, School of Medicine, Johns Hopkins University, Baltimore, Maryland, USA

[4]Department of International Health, Johns Hopkins Bloomberg School of Public Health, Baltimore, Maryland, USA

[5]Department of Global Public Health, Karolinska Institutet, Stockholm, Sweden

[6]Department of Paediatrics, Shaheed Suhrawardi Medical College Hospital, Dhaka, Bangladesh

[7]Royal Hospital for Children, Glasgow, UK

[8]Department of Neonatology, Institute of Child and Mother Health, Dhaka, Bangladesh

[9]Centre for Inflammation Research, University of Edinburgh, UK

Corresponding Author:

Ahad Mahmud Khan

The University of Edinburgh, Edinburgh, UK

Email: ahad_mahmud@hotmail.com, A.M.Khan@sms.ed.ac.uk

Preprint: https://doi.org/10.1101/2022.08.08.22278556

For peer review only

For peer review only - http://bmjopen.bmj.com/site/about/guidelines.xhtml

Abstract

Introduction

Manual counting of respiratory rate (RR) in children is challenging for health workers and can result in misdiagnosis of pneumonia. Some novel RR counting devices automate the counting of RR and classification of fast breathing. The absence of an appropriate reference standard to evaluate the performance of these devices is a challenge. If good quality videos could be captured, with RR interpretation from these videos systematically conducted by an expert panel, it could act as a reference standard. This study is designed to develop a video expert panel (VEP) as a reference standard to evaluate RR counting for identifying pneumonia in children.

Methods and analysis

Using a cross-sectional design, we will enrol children aged 0-59 months presenting with suspected pneumonia at different levels of health facilities in Dhaka and Sylhet, Bangladesh. We will video record a physician/health worker counting RR manually and also using an automated RR counter (ChARM) from each child. We will establish a standard operating procedure for capturing quality videos, make a set of reference videos, and train and standardise the VEP members using the reference videos. After that, we will assess the performance of the VEP as a reference standard to evaluate RR counting. We will calculate the mean difference and proportions of agreement within ±2 breaths per minute and create Bland-Altman plots with limits of agreement between VEP members.

Ethics and dissemination

The study protocol was approved by the National Research Ethics Committee of Bangladesh Medical Research Council (BMRC), Bangladesh (registration number: 39315022021) and Edinburgh Medical School Research Ethics Committee (EMREC), Edinburgh, UK (REC Reference: 21-EMREC-040). Dissemination of the study findings will be through conference presentations and publications in peer-reviewed scientific journals.

Keywords

Pneumonia, respiratory rate, fast breathing, reference standard, video expert panel

Word count: 2771

For peer review only - http://bmjopen.bmj.com/site/about/guidelines.xhtml

Strengths and limitations of this study

- Expert paediatricians will provide feedback to develop a standard operating procedure for videography of child chest movements.
- The video expert panel will be trained and standardized using the expert paediatrician-interpreted reference videos.
- Video expert panel members will be masked to respiratory rate counted by each other and to respiratory rate manual counts, and with the automated counter.
- Children with varying severity of illness will be enrolled from different levels of health facilities in Bangladesh.
- Despite the availability of multiple respiratory rate counters (e.g., ChARM, Rad-G, uPM60), only the ChARM device will be used, which is a limitation in this study.

For peer review only

Introduction

Pneumonia is one of the leading causes of mortality in children aged below five years worldwide.[1] Approximately 68 million pneumonia episodes and 0.65 million deaths occur annually in under-five children due to pneumonia.[2] The highest number of deaths due to pneumonia among children below five years were in sub-Saharan Africa, South Asia, and Southeast Asia. There were an estimated 2.7 million episodes of pneumonia in Bangladesh and 21,275 deaths due to childhood pneumonia in 2015.[3]

According to the World Health Organization (WHO) guidelines, pneumonia diagnosis in children for frontline health workers is primarily based on increased respiratory rate (RR) and/or chest indrawing.[4 5] Fast breathing is the most common sign of pneumonia. It is most commonly identified by counting the RR manually.[5] However, manual counting can be challenging for the health workers[6], often leading to incorrect diagnosis and, consequently, inappropriate treatment.[7-9]

Better performing RR diagnostics to support frontline health workers might improve the diagnosis of pneumonia. Three automated RR counting devices are considered suitable for use by health workers, i.e., Children's Automated Respiration Monitor (ChARM)[10 11], Rad-G[12 13] and uPM60[14]. The Philips ChARM converts chest movements detected by accelerometers into a precise breathing count using specially designed algorithms. The device is placed around the child's belly, and it automatically counts the number of respirations for a particular period, calculates RR and classifies fast breathing according to WHO guidelines[10 11]. The usability and acceptability of this device have been tested in a study in Ethiopia[15] and Nepal. [16]

A reference standard is essential to evaluate the performance of RR counters in field settings. However, the absence of an appropriate reference standard is a challenge. Different reference standards have been used in various studies.[17 18] Most of the existing studies have used manual RR count by an expert person (e.g., physician, nurse) as the reference standard.[19] Few studies have been found using automated monitors.[20 21] The possible biases using a human expert's count as the reference standard include difficulty measuring the RR over the same simultaneous period and inconsistencies in human expert RR counting.[6] The automated monitors do not measure chest movements directly, but other variables such as carbon dioxide (CO_2), pulse oximeter signal or photoplethysmogram (PPG), sound etc., are used to extract RR. [19 22-24]

Videography of a child's chest movements and interpretation by the experts could be used as a reference standard. [17 18 25] If quality videos could be recorded and the interpretation of RR from these videos could be systematically conducted by a video expert panel (VEP), it could be an ideal and non-biased reference standard. This study aims to develop a VEP as a reference standard for evaluating RR counting manually and using ChARM in paediatric pneumonia diagnosis. We will establish a standard operating procedure (SOP) for capturing quality videos, make a set of reference videos, and train and standardise the VEP members using the reference videos. The SOP, recorded videos, and evaluation methods could be used for evaluating new RR counting devices.

Study objectives

Primary objectives:

1. To develop a SOP for video recording a child's chest movements for RR interpretation.

For peer review only - http://bmjopen.bmj.com/site/about/guidelines.xhtml

2. To create a set of reference videos for the training and standardisation of the VEP members.
3. To train and standardise the VEP members to interpret RR using the reference videos.
4. To assess the performance of the VEP to evaluate RR counting manually and with ChARM based on videos captured in real-world settings.

Secondary objectives:

1. To assess the accuracy of the ChARM device in counting RR compared to the VEP as the reference.
2. To determine the duration of counting RR using the ChARM device.
3. To explore the potential influence of the ChARM device on RR count compared to standard observation techniques.
4. To assess the agreement of manual RR count by healthcare staff with RR counted by VEP.

Methods and analysis

Study design and settings

This will be a cross-sectional study. First, we will capture videos of child chest movements, develop a SOP of videography, make a set of reference videos and use these reference videos to train and standardize the VEP. We will record these videos from the inpatient department (IPD) of the Institute of Child and Mother Health (ICMH), Dhaka, Bangladesh where children with different severity of illness are admitted. After that, we will use the VEP as a reference standard to evaluate RR counting. The videos will be recorded from different levels of health facilities in Bangladesh. Three community clinics (CCs) and a sub-district hospital (Zakiganj Upazila Health Complex) in Sylhet and ICMH in Dhaka will be selected. CCs are the lower-level health facility in Bangladesh staffed by community healthcare providers (CHCP).[26] Patients from the CCs are referred to sub-district hospitals. Patients often go to sub-district hospitals directly.[27] The study started on 6 December 2021 and is planned to be completed by December 2022.

Study population

Inclusion criteria:

i. Age <2 months presenting with any illness
ii. Age 2-59 months presenting with cough and/or difficulty breathing

Exclusion criteria:

i. Presence of any danger sign (unable to drink or feed, vomit everything, convulsions, lethargic or unconscious)
ii. The parents are unwilling to provide consent

Study procedures

Enrolment and consenting:

For peer review only - http://bmjopen.bmj.com/site/about/guidelines.xhtml

In the hospital, a physician will screen all children who will visit the outpatient clinic (sub-district hospital) or are already admitted to the inpatient department (ICMH) and enrol eligible children. In the CC, the CHCP will screen every child who will visit his/her CC. If a child becomes eligible, the CHCP will enrol the child and contact research staff who will visit the CC immediately and will complete data collection. Informed written consent will be obtained from the child's mother, father, or other available caregiver before data collection.

Developing SOP of videography:

Research staff will record videos of child chest movements from 30 children. The Canon EOS M50 camera[28] will be used for recording videos. If a good source of natural light is not available, we will use a light-emitting diode (LED) lamp for lighting. The child's clothes will be removed from the lower end of the neck through the umbilicus so that the chest and belly are exposed. The child's face or any other identifiable part will not be recorded. The videography procedure (e.g., camera positions, angles and levels of light, use of tripod etc.) will be varied between children depending on the child and environmental factors (e.g., child condition, position, adequate exposure, place of assessment etc.). Video recording will be done both when counting RR manually twice and using the ChARM device twice.

Once the child is calm, the research staff will start video recording, and the physician will tap the microphone with his/her finger and begin counting RR. The physician will count RR for one minute and tap the microphone again when counting is finished, and the research staff will stop the recording. After that, the physician will attach the ChARM device, and the research staff will start video recording. Once the child is calm, the physician will start the ChARM device and also begin counting RR manually, and the video recording will be done in parallel to the physician's use of ChARM like before. The physician will count the total number of breaths until ChARM completes its counting. The ChARM device will show a green or red signal when it finishes its count. The video recording will continue until the device is done. The time taken by the ChARM device to get the RR will be recorded. A maximum of 3 minutes will be allowed for the ChARM to register a reading or display an error message. Otherwise, it will be documented as a failed attempt. The physician will make a maximum of three attempts for each manual count and ChARM count.

Each video file will be saved in the camera with a file name. The research staff will note the file name in the data collection sheet. Then the videos will be downloaded from the camera and saved on a password-protected computer and the videos will be deleted the camera. After that he will edit the video by removing additional portions of the recording, removing sound or identifiable features, and reducing file size. Video Editing Software (Adobe Premiere Pro) will be used for video editing.

There will be two expert paediatricians (i.e., medical practitioners having a post-graduate degree in paediatrics and specialized in treating sick children). These videos will be sent to them for their feedback on video quality (decision of using tripod, camera positions, angles, light, or other issues) and to count RR. Their feedback will be incorporated, and the video recording procedures will be modified accordingly. A SOP for videography will be developed to maintain consistency across all study hospitals/clinics.

Figure 1 shows the schematic diagram of developing the SOP for videography.

For peer review only - http://bmjopen.bmj.com/site/about/guidelines.xhtml

Making a set of reference videos:

Following the development of the SOP, videos will be recorded from 50 children using this standardised procedure. A physician will count the RR manually once and with the ChARM device once, and video recording will be done simultaneously. The same procedure described in Figure 1 will be followed. If the physician fails in their first attempt to have a satisfactory recording, they will make two more attempts for each manual and ChARM counts. The reason for the attempted failure will be documented. The condition of the child during RR measurement will also be noted.

There will be three expert paediatricians. Each video will randomly be allocated to two of the paediatricians. If both paediatricians disagree, i.e., disagreement in readability or difference of RR >±2 breaths per minute (bpm), then the video will be sent to the third paediatrician. If any two paediatricians agree, i.e., agreement in interpretability and difference of RR ≤±2 bpm, the videos will be considered reference videos. The mean RR count of paediatricians in agreement will be considered as the final RR count.

Figure 2 presents the schematic diagram of making a set of reference videos.

Training and standardisation of VEP members:

Six local physicians with a MBBS degree will be invited for training and standardisation. The training session will be conducted using an online platform. The physicians will be trained on counting RR using videos of known RR counts with different level of breathing from reference videos. Training will be provided following the WHO Integrated Management of Childhood Illness (IMCI) guidelines.[4 5] The Principal Investigator (PI) will conduct the training sessions. After completion of training, they will be evaluated individually in a blinded manner. Twenty videos with different level of breathing will be assigned to each VEP member from the reference videos. The RR interpreted by each VEP member will be considered accurate if this is within ±2bpm of the RR interpreted by expert paediatricians. Those physicians who achieve a pass mark (at least 80%) in the evaluation will be considered to work as VEP members in the study.

Evaluating the performance of VEP:

The performance of VEP will be evaluated using the videos recorded in different levels of health facilities (CCs, sub-district hospital and ICMH). RR of each child will be assessed twice, i.e., manually and using ChARM by the CHCP in the CC and by a physician in the hospital (as described in Figure 1). The videos will be recorded following the SOP. Each video will be randomly assigned to two VEP members to interpret the RR. If there is an agreement of being uninterpretable, the videos will be excluded. If the RR is ≤±2 bpm between both members, their average RR will be considered final. However, if there is a disagreement in interpretability or the difference of the RR count is >2 bpm between the first two members, the video will be sent to a third-panel member. If there are inconsistencies among all three members (if the RR is not ≤2 bpm between any two members), the videos will be sent to a fourth-panel member. If there is agreement between the fourth-panel member and any other member who read the video earlier, their average RR will be considered final. However, if there is no agreement between any two panel members, the videos will be excluded from the analysis (Figure 3).

For peer review only - http://bmjopen.bmj.com/site/about/guidelines.xhtml

Quality control measures:

The videos will be checked periodically if they adhere to the SOP to ensure quality by the PI. Any quality issues will be reported, and corrective feedback will be provided. The expert paediatricians will view a 10% random sample of videos in which they are blinded to the panel's final classification. The panel's overall performance will be evaluated using the expert's interpretation as the reference. Lastly, to monitor individual member interpretation reproducibility, all members will randomly be reassigned 20% of images they interpreted previously (at least one week earlier) to check intra-reader agreement.

Development of a web-based automated system:

A web-based automated system will be developed. This system will use the Hypertext Preprocessor (PHP) platform for the user interface and the Structured Query Language (SQL) platform for the database. The purpose of developing this system is an automated distribution of the video recordings among the expert paediatricians and VEP members, the input of readings by the members and the generation of automated reports. The system will be password-protected and connected to a secured server. The expert paediatricians and VEP members will use this program. Each will need to log in with their user ID and password. Access to each expert paediatrician and VEP member will be limited to what they are assigned. When logged in, they will see the list of recordings assigned to them. They will open the video on the computers and interpret the RR. The VEP members will be blinded to other members' readings.

Operational definitions

Interpretability:

The video will be considered 'interpretable' when the expert paediatrician or VEP member will be able to view the child's chest movements and measure RR for the whole duration. On the other hand, a video will be counted as 'uninterpretable' if the physician will not be able to view and count RR for the whole duration of the recording. The videos can be unreadable for various reasons, e.g., wrong position and angle of the camera, inappropriate focus, poor lighting, inadequate exposure or if the child is agitated, moving, crying etc.

Agreement and disagreement:

The agreement will be defined when two expert paediatricians or VEP members interpret the video as

- uninterpretable or
- interpretable and the difference between RR count ≤±2 bpm.

Disagreement is when

- one expert paediatrician/VEP member interprets the video as interpretable, and another interprets it as unreadable or
- the difference between their RR count is >2 bpm.

Data collection and storage

Field data (age, sex, RR, child condition during assessment) will be collected in paper forms. After entry, data will be transferred to a password-protected server (SQL Server 2008 R2) located in Dhaka, Bangladesh, in real-time using internet connectivity. Recorded videos will

For peer review only - http://bmjopen.bmj.com/site/about/guidelines.xhtml

be transferred to encrypted OneDrive/Dropbox cloud storage. The VEP members will interpret RR from recorded videos using the web-based automated software, and the data will also be stored on the server in real-time. De-identified data will be transferred and stored on the DataShare server at the University of Edinburgh, UK.

Sample size calculation

The videos from the first 30 children will be used to establish the best procedure for capturing videos. This number was purposively chosen as a qualitative assessment of the videos will be done. We assume that 30 children would be enough to develop the SOP. However, we will consider recruiting more if after 30 children a consistent SOP has not been finalized. After finalization of the SOP, the videos from 50 children will be used for making a set of reference videos to train and standardize VEP. We did not use any statistical formula to calculate this sample size. These children will be recruited from the IPD of a hospital. Based on our experience in this setting, we assume that we will get a significant number of children with fast breathing. However, we will consider recruiting further children if our target has not been achieved. To evaluate the performance of VEP, Bland-Altman's statistical methods for assessing agreement between two methods of clinical measurement are used.[29 30] A total of 226 interpretable videos of each method (manual and ChARM) are required, which will provide 90% power to assess agreement in RR counts between two VEP members, considering type-I error α=0.05, expected mean difference ±0.5, expected SD 1.5 and maximum allowed difference 4. Assuming 80% interpretability of the videos and 20% failure to record videos, the required number of children is about 350.

Statistical analysis

To standardize the VEP members, we will estimate the mean difference of RR counts and the percent agreement within ±2bpm between each VEP member and expert paediatricians. We will consider expert paediatricians' counts as the gold standard. To assess the performance of VEP members, we will produce Bland-Altman plots[29] to assess the agreement in RR counts and calculate the percent agreement between primary panel members. We will calculate mean difference of RR counts and the percent agreement within ±2bpm to assess the intra-reader agreement of each VEP member. We will use Cohen's kappa statistic to estimate the inter-reader agreement of identifying fast breathing between panel members. We will also measure the mean difference of RR counts and the percent agreement within ±2bpm between each VEP member and expert paediatricians from QC data. To evaluate the accuracy of the ChARM device and manual counts, we will produce Bland-Altman plots to assess the agreement in RR counts of ChARM counts, and manual counts with VEP counts. We will measure the mean difference of RR counts and the percent agreement within ±2bpm. We will also use Cohen's kappa statistic to assess the agreement in identifying fast breathing between these methods. The mean time to measure the RR by the ChARM device will be assessed. To assess the potential influence of the ChARM device on the RR of the children, we will calculate the mean difference of RR between the first and second measurements interpreted by the VEP.

Ethics and dissemination

Ethical approval was obtained from the National Research Ethics Committee of Bangladesh Medical Research Council (BMRC), Bangladesh (Registration Number: 39315022021), and

For peer review only - http://bmjopen.bmj.com/site/about/guidelines.xhtml

Edinburgh Medical School Research Ethics Committee (EMREC), Edinburgh, UK (REC Reference: 21-EMREC-040). Informed written consent will be taken from the parent or guardian of each child. Dissemination will be through conference presentations and publications in peer-reviewed journals. Anonymised data files will also be stored securely in the DataStore repository at the University of Edinburgh, UK and will be shared after the publication the main paper.

Patient and public involvement

Patients and/or the public were not involved in this research's design, conduct, reporting, or dissemination plans.

Acknowledgments

The authors are grateful for the support and contributions of Dr Arunangshu Dutta Roy, Asim Nehal, Rizouan Ur Rashid, Rakib Bhuiyan, Dr Nusrat Sharmin Asma, Dr Farjana Hossain, Dr Muhammad Shariful Islam, Dr Md. Jahurul Islam, and the Ministry of Health and Family Welfare, Government of Bangladesh. The authors also thank RESPIRE collaboration for their contribution in bringing the manuscript to its final shape. The RESPIRE collaboration comprises the UK Grant holders, Partners and research teams as listed on the RESPIRE website (www.ed.ac.uk/usher/respire) including Linda Bauld.

Footnotes

Author Contributions

AMK and HC conceptualised and designed this study. HC, SC, AHB, EDM and TS provided mentorship to AMK. HC, SC, EDM and CK critically reviewed the study design. AMK, SA, MMR will be involved in project management. KN, RMS and AA will review the videos and provide feedback to video quality. NHC and MSI will be responsible for data management. AMK drafted the manuscript, and all authors critically reviewed and approved the final manuscript before submission.

Funding

This research was funded by the UK National Institute for Health Research (NIHR) (Global Health Research Unit on Respiratory Health (RESPIRE); 16/136/109) using UK aid from the UK Government to support global health research. The views expressed in this publication are those of the author(s) and not necessarily those of the NIHR or the UK Government.

Competing interests

None declared.

References

1. Liu L, Oza S, Hogan D, et al. Global, regional, and national causes of under-5 mortality in 2000-15: an updated systematic analysis with implications for the Sustainable Development Goals. *Lancet* 2016;388(10063):3027-35. doi: 10.1016/s0140-6736(16)31593-8 [published Online First: 2016/11/15]
2. GBD 2016. Estimates of the global, regional, and national morbidity, mortality, and aetiologies of lower respiratory infections in 195 countries, 1990-2016: a systematic analysis for the Global Burden of Disease Study 2016. *Lancet Infect Dis* 2018;18(11):1191-210. doi: 10.1016/s1473-3099(18)30310-4 [published Online First: 2018/09/24]

For peer review only - http://bmjopen.bmj.com/site/about/guidelines.xhtml

3. GBD 2015. Estimates of the global, regional, and national morbidity, mortality, and aetiologies of lower respiratory tract infections in 195 countries: a systematic analysis for the Global Burden of Disease Study 2015. *Lancet Infect Dis* 2017;17(11):1133-61. doi: 10.1016/s1473-3099(17)30396-1 [published Online First: 2017/08/28]
4. World Health Organization. IMCI information package. Integrated Management of Childhood Illness (IMCI). Geneva, 1999.
5. World Health Organization. Integrated Management of Childhood Illness: Chart Booklet. *World Health Organization: Geneva* 2014
6. Khan AM, O'Donald A, Shi T, et al. Accuracy of non-physician health workers in respiratory rate measurement to identify paediatric pneumonia in low-and middle-income countries: A systematic review and meta-analysis. *J Glob Health* 2022;12
7. Kallander K, Tomson G, Nsabagasani X, et al. Can community health workers and caretakers recognise pneumonia in children? Experiences from western Uganda. *Transactions of the Royal Society of Tropical Medicine and Hygiene* 2006;100(10):956-63. doi: 10.1016/j.trstmh.2005.11.004 [published Online First: 2006/02/04]
8. Mukanga D, Babirye R, Peterson S, et al. Can lay community health workers be trained to use diagnostics to distinguish and treat malaria and pneumonia in children? Lessons from rural Uganda. *Tropical medicine & international health : TM & IH* 2011;16(10):1234-42. doi: 10.1111/j.1365-3156.2011.02831.x [published Online First: 2011/07/15]
9. Miller NP, Amouzou A, Tafesse M, et al. Integrated community case management of childhood illness in Ethiopia: implementation strength and quality of care. *The American journal of tropical medicine and hygiene* 2014;91(2):424-34. doi: 10.4269/ajtmh.13-0751 [published Online First: 2014/05/07]
10. Shah R, Jenda G, Lwesha V, et al. An integrated diagnostic device for neonatal sepsis and childhood pneumonia. *Journal of public health in Africa* 2018;9(1):780. doi: 10.4081/jphia.2018.780 [published Online First: 2018/08/07]
11. Philips. Philips Introduces New Diagnostic Device to Help Prevent Childhood Pneumonia in Low-resource Countries 2019 [Available from: file:///C:/Users/Ahad/Downloads/Backgrounder%20-%20Philips%20Children%E2%80%99s%20Automated%20Respiration%20Monitor%20(1).pdf accessed 18 November 2019.
12. Masimo. Masimo Introduces Rad-G™ Pulse Oximeter 2017 [Available from: http://masimocorp.gcs-web.com/news-releases/news-release-details/masimo-introduces-rad-gtm-pulse-oximeter accessed viewed 07 November 2018.
13. Trademarkia. RAD-G Trademark Information 2017 [Available from: https://www.trademarkia.com/radg-87345900.html accessed 07 November 2018.
14. Utech. uPM60 Handheld Vital Signs Monitor 2020 [Available from: http://www.chinautech.com/cn/col.jsp?id=161 accessed 10 May 2020.
15. Ward C, Baker K, Smith H, et al. Usability and acceptability of an automated respiratory rate counter to assess children for symptoms of pneumonia: a prospective, cohort study in Ethiopia. *Acta paediatrica (Oslo, Norway : 1992)* 2019 doi: 10.1111/apa.15074 [published Online First: 2019/10/23]
16. Källander K, Ward C, Smith H, et al. Usability and acceptability of an automated respiratory rate counter to assess childhood pneumonia in Nepal. *Acta paediatrica (Oslo, Norway : 1992)* 2020;109(6):1207-20. doi: 10.1111/apa.15108 [published Online First: 2019/11/26]
17. Selecting a reference standard for evaluating respiratory rate devices to diagnose symptoms of pneumonia in children under 5: Lessons learned from resource-poor settings in Sub-Saharan Africa and Asia. American Journal of Tropical Medicine and Hygiene; 2017. Amer Soc Trop Med & Hygiene 8000 Westpart Dr, Ste 130, Mclean, VA 22101 USA.

For peer review only - http://bmjopen.bmj.com/site/about/guidelines.xhtml

18. Ansermino JM, Dumont G, Ginsburg AS. How Uncertain Is Our Reference Standard for Respiratory Rate Measurement? *Am J Respir Crit Care Med* 2019;199(8):1036-37. doi: 10.1164/rccm.201812-2266LE [published Online First: 2019/01/24]
19. Ginsburg AS, Lenahan JL, Izadnegahdar R, et al. A Systematic Review of Tools to Measure Respiratory Rate in Order to Identify Childhood Pneumonia. *Am J Respir Crit Care Med* 2018;197(9):1116-27. doi: 10.1164/rccm.201711-2233CI [published Online First: 2018/02/24]
20. Baker K, Alfven T, Mucunguzi A, et al. Performance of Four Respiratory Rate Counters to Support Community Health Workers to Detect the Symptoms of Pneumonia in Children in Low Resource Settings: A Prospective, Multicentre, Hospital-Based, Single-Blinded, Comparative Trial. *EClinicalMedicine* 2019;12:20-30. doi: 10.1016/j.eclinm.2019.05.013 [published Online First: 2019/08/08]
21. Becker C, Achermann S, Rocque M, et al. Camera-based measurement of respiratory rates is reliable. *Eur J Emerg Med* 2018;25(6):416-22. doi: 10.1097/mej.0000000000000476 [published Online First: 2017/06/03]
22. Charlton PH, Bonnici T, Tarassenko L, et al. An assessment of algorithms to estimate respiratory rate from the electrocardiogram and photoplethysmogram. *Physiol Meas* 2016;37(4):610-26. doi: 10.1088/0967-3334/37/4/610 [published Online First: 2016/03/31]
23. Li W, Liu J, Yu L. [Study on Respiratory Rate Monitoring Method Based on Breath Sounds]. *Zhongguo Yi Liao Qi Xie Za Zhi* 2018;42(6):391-94. doi: 10.3969/j.issn.1671-7104.2018.06.001 [published Online First: 2018/12/19]
24. Daw W, Kingshott R, Saatchi R, et al. Medical devices for measuring respiratory rate in children: a review. *Journal of Advances in Biomedical Engineering and Technology* 2016;3:21-27.
25. Stratil AS, Ward C, Habte T, et al. Evaluating the Interrater Agreement and Acceptability of a New Reference Tool for Assessing Respiratory Rate in Children under Five with Cough and/or Difficulty Breathing. *J Trop Pediatr* 2021;67(2) doi: 10.1093/tropej/fmab046
26. Karim RM, Abdullah MS, Rahman AM, et al. Identifying role of perceived quality and satisfaction on the utilization status of the community clinic services; Bangladesh context. *BMC Health Serv Res* 2016;16:204. doi: 10.1186/s12913-016-1461-z [published Online First: 2016/06/28]
27. Ahmed S, Siddique AK, Iqbal A, et al. Causes for hospitalizations at upazila health complexes in Bangladesh. *J Health Popul Nutr* 2010;28(4):399-404. doi: 10.3329/jhpn.v28i4.6047 [published Online First: 2010/09/10]
28. Canon. Canon EOS M50 [Available from: https://www.canon-europe.com/cameras/eos-m50/ accessed 21 June 2021 2020.
29. Bland JM, Altman DG. Statistical methods for assessing agreement between two methods of clinical measurement. *Lancet* 1986;1(8476):307-10.
30. Lu MJ, Zhong WH, Liu YX, et al. Sample Size for Assessing Agreement between Two Methods of Measurement by Bland-Altman Method. *Int J Biostat* 2016;12(2) doi: 10.1515/ijb-2015-0039 [published Online First: 2016/11/14]

Figure 1: Developing SOP of videography

Figure 2: Making a set of reference videos

Figure 3: Respiratory rate interpretation from video recording by video expert panel

For peer review only - http://bmjopen.bmj.com/site/about/guidelines.xhtml

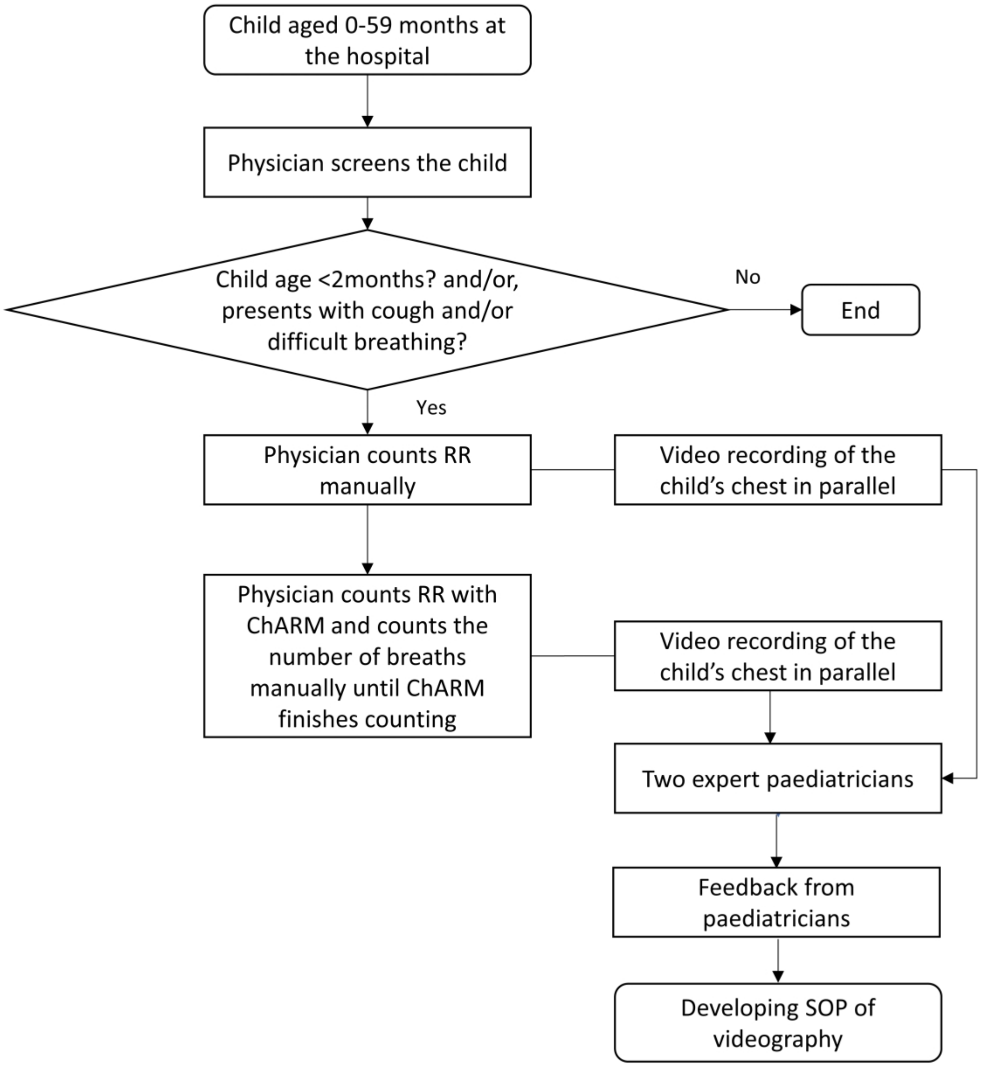

Developing SOP of videography

456x497mm (236 x 236 DPI)

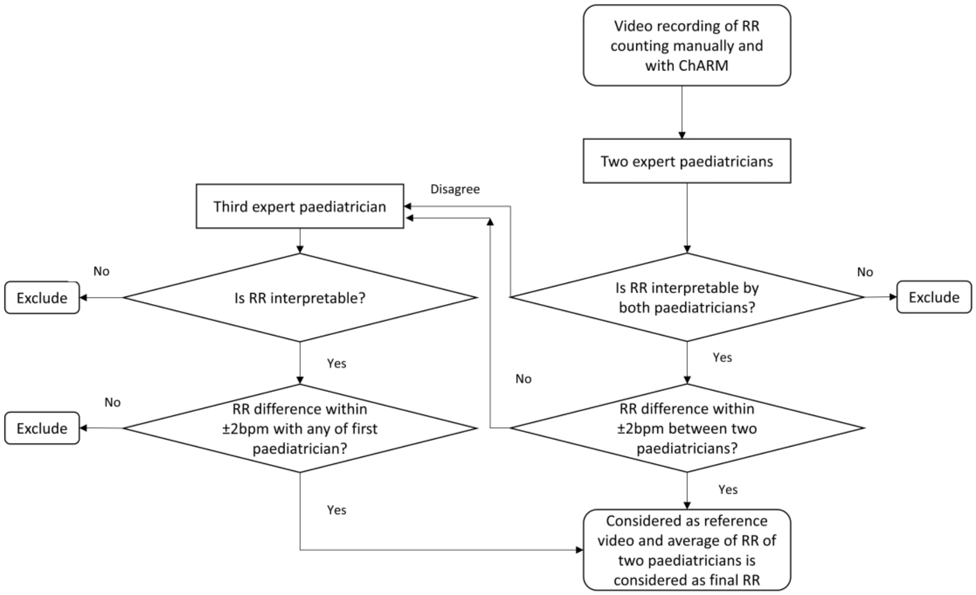

Making a set of reference videos

719x433mm (236 x 236 DPI)

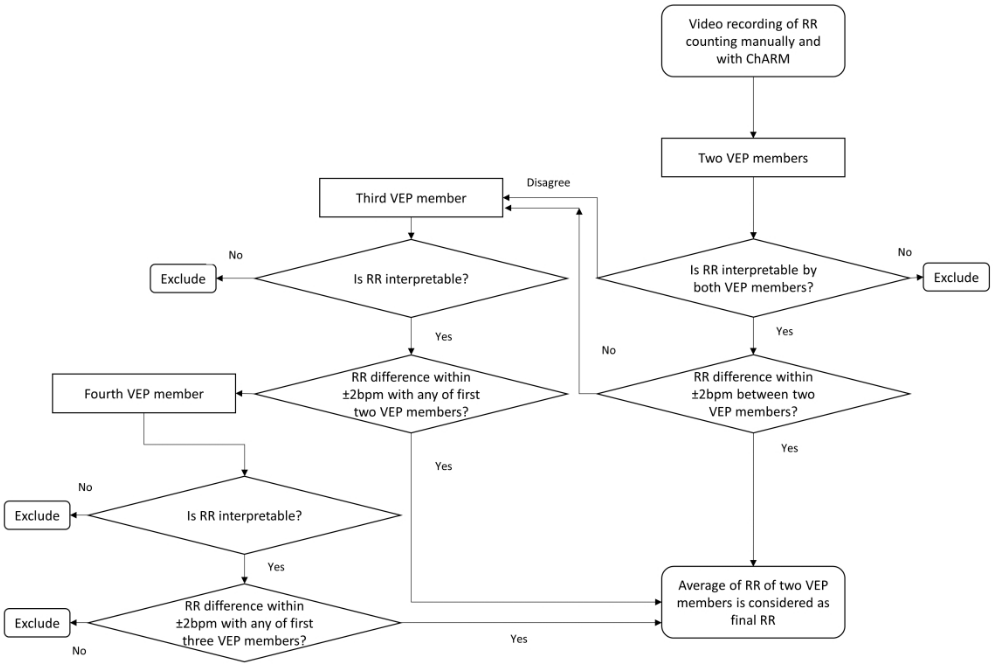

Figure 3 Respiratory rate interpretation from video recording by video expert panel

813x557mm (118 x 118 DPI)

STROBE Statement—Checklist of items that should be included in reports of ***cross-sectional studies***

	Item No	Recommendation	Page No
**Title and abstract**	1	(*a*) Indicate the study's design with a commonly used term in the title or the abstract	1, 2
		(*b*) Provide in the abstract an informative and balanced summary of what was done and what was found	2
**Introduction**			
Background/rationale	2	Explain the scientific background and rationale for the investigation being reported	4
Objectives	3	State specific objectives, including any prespecified hypotheses	4,5
**Methods**			
Study design	4	Present key elements of study design early in the paper	5
Setting	5	Describe the setting, locations, and relevant dates, including periods of recruitment, exposure, follow-up, and data collection	5, 8
Participants	6	(*a*) Give the eligibility criteria, and the sources and methods of selection of participants	5
Variables	7	Clearly define all outcomes, exposures, predictors, potential confounders, and effect modifiers. Give diagnostic criteria, if applicable	8
Data sources/ measurement	8*	For each variable of interest, give sources of data and details of methods of assessment (measurement). Describe comparability of assessment methods if there is more than one group	*5-8*
Bias	9	Describe any efforts to address potential sources of bias	N/A
Study size	10	Explain how the study size was arrived at	8-9
Quantitative variables	11	Explain how quantitative variables were handled in the analyses. If applicable, describe which groupings were chosen and why	9
Statistical methods	12	(*a*) Describe all statistical methods, including those used to control for confounding	9
		(*b*) Describe any methods used to examine subgroups and interactions	9
		(*c*) Explain how missing data were addressed	N/A
		(*d*) If applicable, describe analytical methods taking account of sampling strategy	8
		(*e*) Describe any sensitivity analyses	N/A
**Results**			
Participants	13*	(a) Report numbers of individuals at each stage of study—eg numbers potentially eligible, examined for eligibility, confirmed eligible, included in the study, completing follow-up, and analysed	N/A
		(b) Give reasons for non-participation at each stage	N/A
		(c) Consider use of a flow diagram	N/A
Descriptive data	14*	(a) Give characteristics of study participants (eg demographic, clinical, social) and information on exposures and potential confounders	N/A
		(b) Indicate number of participants with missing data for each variable of interest	N/A
Outcome data	15*	Report numbers of outcome events or summary measures	N/A
Main results	16	(*a*) Give unadjusted estimates and, if applicable, confounder-adjusted estimates and their precision (eg, 95% confidence interval). Make clear which confounders were adjusted for and why they were included	N/A

For peer review only - http://bmjopen.bmj.com/site/about/guidelines.xhtml

For peer review only

		(*b*) Report category boundaries when continuous variables were categorized	N/A
		(*c*) If relevant, consider translating estimates of relative risk into absolute risk for a meaningful time period	N/A
Other analyses	17	Report other analyses done—eg analyses of subgroups and interactions, and sensitivity analyses	N/A
**Discussion**			
Key results	18	Summarise key results with reference to study objectives	N/A
Limitations	19	Discuss limitations of the study, taking into account sources of potential bias or imprecision. Discuss both direction and magnitude of any potential bias	N/A
Interpretation	20	Give a cautious overall interpretation of results considering objectives, limitations, multiplicity of analyses, results from similar studies, and other relevant evidence	N/A
Generalisability	21	Discuss the generalisability (external validity) of the study results	N/A
**Other information**			
Funding	22	Give the source of funding and the role of the funders for the present study and, if applicable, for the original study on which the present article is based	9

*Give information separately for exposed and unexposed groups.

Note: An Explanation and Elaboration article discusses each checklist item and gives methodological background and published examples of transparent reporting. The STROBE checklist is best used in conjunction with this article (freely available on the Web sites of PLoS Medicine at http://www.plosmedicine.org/, Annals of Internal Medicine at http://www.annals.org/, and Epidemiology at http://www.epidem.com/). Information on the STROBE Initiative is available at www.strobe-statement.org.

For peer review only

For peer review only - http://bmjopen.bmj.com/site/about/guidelines.xhtml
